# Supplementary material for: Understanding park visitors’ soundscape perception using subjective and objective measurement
Source: PeerJ. 2024 Jan 31;12:e16592. doi: 10.7717/peerj.16592 (PMC10838067; doi:10.7717/peerj.16592)
Supplement: Supplemental Information 3 [file peerj-12-16592-s003.docx]

**Muir Woods National Monument**

**Visitor Study**

1. Visitors have different reasons for visiting Muir Woods National Monument. Please rate the importance of each of the following reasons for your visit to Muir Woods National Monument today. *Please mark only one response for each item.*

| **Importance…** | **Not Relevant** | **Not at all Important** | **Slightly Important** | **Moderately Important** | **Very Important** | **Extremely Important** |
| --- | --- | --- | --- | --- | --- | --- |
| To experience a sense of connection with nature | □ | □ | □ | □ | □ | □ |
| To experience the diversity of the natural world | □ | □ | □ | □ | □ | □ |
| To enjoy the natural quiet and sounds of nature | □ | □ | □ | □ | □ | □ |
| To give my mind a rest | □ | □ | □ | □ | □ | □ |
| To get away from the usual demands of life | □ | □ | □ | □ | □ | □ |
| To get away from the noise back home | □ | □ | □ | □ | □ | □ |
| To develop your skills and abilities | □ | □ | □ | □ | □ | □ |
| To improve your skills | □ | □ | □ | □ | □ | □ |
| To do something with your family | □ | □ | □ | □ | □ | □ |
| To be with friends | □ | □ | □ | □ | □ | □ |
| To experience wildlife in nature | □ | □ | □ | □ | □ | □ |
| To photograph wildlife | □ | □ | □ | □ | □ | □ |
| Seeing the redwoods | □ | □ | □ | □ | □ | □ |
| Appreciating the scenic beauty | □ | □ | □ | □ | □ | □ |
| Experiencing solitude | □ | □ | □ | □ | □ | □ |
| Getting some exercise | □ | □ | □ | □ | □ | □ |
| Learning about nature | □ | □ | □ | □ | □ | □ |
| Enjoying the peace and quiet | □ | □ | □ | □ | □ | □ |
| Hearing the sounds of nature | □ | □ | □ | □ | □ | □ |

1. Based on your experience today, about how many **different types** of birds would you say are in the trail corridor? *Please mark one response.*

□ 0-3 different types of birds

□ 4-7 different types of birds

□ 8-11 different types of birds

□ 12-15 different types of birds

□ More than 15 different types of birds

3. If you heard bird song today, how would you rate the diversity of the bird song chorus? *Please mark one response.*

| Not at All Diverse | A Little Diverse | Moderately Diverse | Highly Diverse | Extremely Diverse | I did not hear bird song |
| --- | --- | --- | --- | --- | --- |
| □ | □ | □ | □ | □ | □ |

4. Visitors hear a lot of sounds, including natural sounds and human-made sounds. Based on your experience today, how would you rate the pleasantness of the soundscape? *Please mark one response.*

| Very Unpleasant | Moderately Unpleasant | Slightly Unpleasant | Slightly Pleasant | Moderately Pleasant | Very Pleasant |
| --- | --- | --- | --- | --- | --- |
| □ | □ | □ | □ | □ | □ |

5. Based on your experience today, how well were you able to hear natural sounds? *Please mark one response.*

□ Almost always clearly without interference from human-made sound

□ Usually clearly without interference from human-made sound

□ Sometimes clearly without interference from human-made sound

□ Usually with interference from human-made sound

□ Almost always with interference from human-made sound

6. Please check the box corresponding with how well you agree or disagree. *Please mark only one response for each item.*

|  | **Strongly Disagree** | **Disagree** | **Slightly Disagree** | **Slightly Agree** | **Agree** | **Strongly Agree** |
| --- | --- | --- | --- | --- | --- | --- |
| I am sensitive to noise. | □ | □ | □ | □ | □ | □ |
| I find it hard to relax in a place that's noisy. | □ | □ | □ | □ | □ | □ |
| I get mad at people who make noise that keeps me from falling asleep or getting work done. | □ | □ | □ | □ | □ | □ |
| I get annoyed when my neighbors are noisy. | □ | □ | □ | □ | □ | □ |
| I get used to most noises without much difficulty. | □ | □ | □ | □ | □ | □ |

| 7. How crowded did you feel on the trail today? *Please select one number.* | | | | | | | | | |
| --- | --- | --- | --- | --- | --- | --- | --- | --- | --- |
| 1 | 2 | 3 | 4 | 5 | 6 | 7 | 8 | 9 | |
| Not crowded at all | | Slightly crowded | |  | Moderately crowded | | Extremely crowded | | |

8. Including this visit, how many times have you visited Muir Woods National Monument?

Approximate number of visits: _____________

9. Approximately how many hours did you spend in Muir Woods National Monument today?

Approximately _____________hours

10. How many adults and how many children were in your personal group (spouse, family, friends) during this trip to Muir Woods National Monument today? *Please provide a number.*

# of Adults (Age 16 or older) _______ # of Children (Age 15 or younger) _______

11. How would you describe your group?

□ Alone

□ Family

□ Friends

□ Family and Friends

□ Organized Group (e.g., club, educational group)

□ Commercial tour group

□ Other (Please specify):______________________

12. What is your gender?

🞎 Male 🞎 Female

13. In what year were you born?

Year Born: __________________

14. Do you live in the United States?

🞎 Yes (What is your zip code? __________)

🞎 No (What country do you live in? ______________________________

15. What is the highest level of formal education you have completed? *Please mark only one response.*

🞎 Some high school

🞎 High school graduate or GED

🞎 Some college, business or trade school

🞎 College, business or trade school graduate

🞎 Some graduate school

🞎 Master’s, doctoral or professional degree
